# Evaluation of machine learning-based classification of clinical impairment and prediction of clinical worsening in multiple sclerosis

**DOI:** 10.1007/s00415-024-12507-w

**Published:** 2024-06-23

**Authors:** Samantha Noteboom, Moritz Seiler, Claudia Chien, Roshan P. Rane, Frederik Barkhof, Eva M. M. Strijbis, Friedemann Paul, Menno M. Schoonheim, Kerstin Ritter

**Affiliations:** 1 MS Center Amsterdam, Anatomy and Neurosciences, Vrije Universiteit Amsterdam, Amsterdam Neuroscience, Amsterdam UMC Location VUmc, Amsterdam, The Netherlands; 2 Department of Psychiatry and Psychotherapy, Charité - Universitätsmedizin Berlin, Corporate Member of Freie Universität Berlin, Humboldt-Universität zu Berlin, Berlin, Germany; 3 Experimental and Clinical Research Center, Charité - Universitätsmedizin Berlin, Corporate Member of Freie Universität Berlin, Humboldt-Universität zu Berlin, Berlin, Germany; 4 Bernstein Center for Computational Neuroscience Berlin, Berlin, Germany; 5 MS Center Amsterdam, Radiology and Nuclear Medicine, Vrije Universiteit Amsterdam, Amsterdam Neuroscience, Amsterdam UMC Location VUmc, Amsterdam, The Netherlands; 6 Centre for Medical Image Computing, Queen Square Institute of Neurology, University College London, London, UK; 7 MS Center Amsterdam, Radiology and Nuclear Medicine, Vrije Universiteit Amsterdam, Amsterdam Neuroscience, Amsterdam UMC Location VUmc, Amsterdam, The Netherlands

**Keywords:** Multiple sclerosis, Machine learning, Magnetic resonance imaging (MRI), Disability prediction, Cognition

## Abstract

**Background:**

Robust predictive models of clinical impairment and worsening in multiple sclerosis (MS) are needed to identify patients at risk and optimize treatment strategies.

**Objective:**

To evaluate whether machine learning (ML) methods can classify clinical impairment and predict worsening in people with MS (pwMS) and, if so, which combination of clinical and magnetic resonance imaging (MRI) features and ML algorithm is optimal.

**Methods:**

We used baseline clinical and structural MRI data from two MS cohorts (Berlin: *n* = 125, Amsterdam: *n* = 330) to evaluate the capability of five ML models in classifying clinical impairment at baseline and predicting future clinical worsening over a follow-up of 2 and 5 years. Clinical worsening was defined by increases in the Expanded Disability Status Scale (EDSS), Timed 25-Foot Walk Test (T25FW), 9-Hole Peg Test (9HPT), or Symbol Digit Modalities Test (SDMT). Different combinations of clinical and volumetric MRI measures were systematically assessed in predicting clinical outcomes. ML models were evaluated using Monte Carlo cross-validation, area under the curve (AUC), and permutation testing to assess significance.

**Results:**

The ML models significantly determined clinical impairment at baseline for the Amsterdam cohort, but did not reach significance for predicting clinical worsening over a follow-up of 2 and 5 years. High disability (EDSS ≥ 4) was best determined by a support vector machine (SVM) classifier using clinical and global MRI volumes (AUC = 0.83 ± 0.07, *p* = 0.015). Impaired cognition (SDMT *Z*-score ≤ −1.5) was best determined by a SVM using regional MRI volumes (thalamus, ventricles, lesions, and hippocampus), reaching an AUC of 0.73 ± 0*.*04 (*p* = 0.008).

**Conclusion:**

ML models could aid in classifying pwMS with clinical impairment and identify relevant biomarkers, but prediction of clinical worsening is an unmet need.

**Supplementary Information:**

The online version contains supplementary material available at 10.1007/s00415-024-12507-w.

## Introduction

Multiple sclerosis (MS) is a chronic inflammatory, demyelinating, and neurodegenerative disease with a heterogeneous and unpredictable disease course [1]. Prognostic biomarkers are urgently needed for monitoring disease progression and optimizing therapeutic strategies [2]. The clinical relevance of magnetic resonance imaging (MRI) for diagnosing and monitoring MS by using inflammatory markers (e.g., white matter (WM) lesion counts) is well established [3, 4]. However, these inflammatory markers have limited explanatory value for determining the severity of symptoms and predicting clinical progression [5, 6]. MRI markers of neurodegeneration, instead, are more closely related to clinical outcomes and thought to be the main driver of irreversible disability [7, 8]. Regional volumetric MRI measures such as deep gray matter (DGM) and cortical gray matter (CGM) volumes have shown the closest associations to motor dysfunctions and cognitive decline [9, 10]. However, the use of regional MRI volumes as predictors of disease progression remains largely unexplored.

Machine learning (ML) strategies have been increasingly applied for prediction in medicine and identifying patients at risk [11]. While traditional statistical techniques can typically handle only a few input variables and are often based on strict assumptions, ML is able to derive complex hidden patterns in high-dimensional data [12]. In MS, various ML approaches have been applied, but predicting disability progression with high accuracy remains challenging [13–15]. These studies used progression on the Expanded Disability Status Scale (EDSS) as a prediction target, since it is the predominant outcome measure for defining disability accumulation and progression in MS [16, 17]. However, the reliability of the EDSS is compromised by a significant measurement error and interrater variability [18]. In addition, it is heavily influenced by ambulatory functioning, while upper-extremity dysfunction and cognitive dysfunction are not adequately measured resulting in a low sensitivity in identifying crucial factors for disease progression [19, 20]. Recent evidence suggests that other outcome measures, such as the Timed 25-Foot Walk Test (T25FW) and the 9-Hole Peg Test (9HPT), as well as composite scores of EDSS, T25FW, and 9HPT (EDSS +), may be more sensitive in capturing disease progression [21, 22]. For cognitive functioning, the Symbol Digit Modalities Test (SDMT) is widely employed in clinical trials, because it is highly sensitive for measuring information processing speed (IPS), the most affected cognitive function in MS [23, 24].

In this study, we aimed to systematically compare the performance of ML approaches for classifying clinical impairment and predicting disease progression in people with MS (pwMS), based on a range of (composite) clinical outcomes (EDSS, T25FW, 9HPT, EDSS + , and SDMT). Our secondary aim was to identify which clinical and MRI markers were most important in determining clinical impairment and predicting worsening defined for each outcome. ML approaches included logistic regression, support vectors machine, gradient boosting, and random forest classifiers. Models were trained on two clinical data sets, one early MS cohort from Berlin with a follow-up after 2 years and a long-standing MS cohort from Amsterdam with a follow-up after 5 years.

## Methods

### Study population

Data were retrospectively collected from the early MS cohort of Berlin, Germany (32 people diagnosed with clinically isolated syndrome (CIS) and 93 pwMS) [25, 26] and people with clinically definite MS from the Amsterdam MS cohort, the Netherlands (330 pwMS) [27, 28]. All included subjects were over the age of 18 and had a clinical assessment and a structural MRI examination available at baseline. Clinical measurements included EDSS, 9HPT, T25FW, and SDMT score. The early MS cohort of Berlin had a 2-year clinical follow-up available for all 125 included subjects. For Amsterdam, a 5-year clinical follow-up was available for 225/330 included subjects. The institutional ethics review boards of both institutions (Amsterdam UMC, Amsterdam and Charité, Berlin) approved the study protocol and subjects gave written informed consent prior to participation.

### MRI acquisition

All subjects underwent a 3T MRI examination including the following pulse sequences: 3D T1-weighted (3D-T1) and 3D fluid-attenuated inversion recovery (3D-FLAIR). The scanning protocol in Berlin included a 3D-T1 magnetization prepared rapid acquisition gradient echo sequence (1*.*0 × 1*.*0 × 1*.*0 mm resolution, repetition time (TR) = 1900 ms, echo time (TE) = 3.03 ms, inversion time (TI) = 900 ms, flip angle = 9°) and a 3D-FLAIR sequence (1*.*0 × 1*.*0 × 1*.*0 mm resolution, TR = 6000 ms, TE = 388 ms, TI = 2100 ms), using a Tim Trio scanner (Siemens Medical Systems, Erlangen, Germany). The scanning protocol in Amsterdam included a 3D-T1 fast-spoiled gradient-echo sequence (1 × 0*.*9 × 0*.*9 mm resolution, TR = 7.8 ms, TE = 3 ms, TI = 450 ms, flip angle = 12°) and a 3D-FLAIR sequence (1*.*2 × 1*.*0 × 1*.*0 mm resolution, TR = 8000 ms, TE = 125 ms, TI = 2350 ms), using a GE Signa HDxt scanner (Milwaukee, WI).

### MRI processing

T2-lesion volumes (T2LV) were determined on 3D-FLAIR. In Berlin, lesions were manually segmented using ITK-SNAP (www.itksnap.org) by two expert MRI technicians [26]. In Amsterdam, lesions were automatically segmented using a *k*-nearest neighbor algorithm and visually checked [28–30]. To reduce lesion-associated brain tissue segmentation bias, lesions were filled with values approximating normal WM on 3D-T1 [31]. Whole-brain, CGM, and DGM segmentations were derived for both centers using the FreeSurfer 7.1.1 (http://surfer.nmr.mgh.harvard.edu/) recon-all pipeline on lesion filled 3D-T1. Subsequently, the cortical surface of each subject was parcellated into 210 regions using the Brainnetome Atlas (BNA) [32]. The volumes of the left and right regions were averaged to decrease the number of input features without losing too much anatomical information, resulting in 5 global volumes (whole-brain volume (WBV), CGM volume, DGM volume, lateral ventricular volume (LVV), cortical cerebellum volume), 105 CGM regional volumes, and seven DGM regional volumes (thalamus, accumbens, putamen, caudate, pallidum, amygdala, hippocampus). All volumes, except for T2LV, were corrected for head size by dividing the volume by the estimated total intracranial volume (eTIV).

### Overview of machine learning approach

**Fig. 1:**
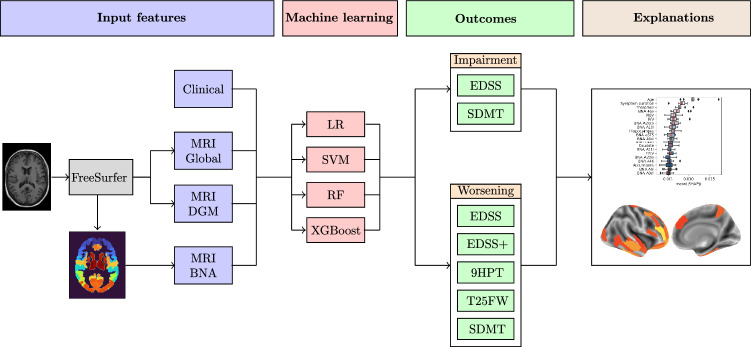
Overview of the machine learning (ML) approach. Input features for ML included different sets of clinical variables and MRI volumes. Different ML approaches were evaluated to classify clinical impairment and predict clinical worsening. Shapley additive explanations were applied to identify the most important clinical and MRI features for classification and prediction. *DGM* deep gray matter; *BNA* Brainnetome Atlas; *LR* logistic regression; *SVM* support vector machine; *RF* random forest; *XGBoost* eXtreme Gradient Boosting; *EDSS* Expanded Disability Status Scale; *SDMT* Symbol Digit Modalities Test; *9HPT* 9-Hole Peg Test; *T25FW* Timed 25-Foot Walk Test

Based on different sets of clinical and MRI features, we trained different ML algorithms for classifying clinical impairment and predicting clinical worsening. The input features included demographic (age, sex) and clinical information (symptom duration, MS subtype, use of disease-modifying therapy (DMT)) as well as structural MRI volumes. The outcome measures for classifying clinical impairment included EDSS (cutoff ≥ 4) to define high disability or SDMT (*Z*-score ≤ −1.5) to define cognitive impairment. Clinical worsening was evaluated over a follow-up of 2 years (Berlin) and 5 years (Amsterdam), based on EDSS, 9HPT, T25FW, or SDMT scores. The significance of classification and prediction models was assessed with permutation testing and the most important clinical and MRI features were determined using Shapley additive explanations (SHAP). See Fig. 1 for an overview of the machine learning approach.

### Clinical impairment

Clinical impairment at baseline was defined using the EDSS or SDMT. PwMS were classified as having a low or high disability based on an EDSS ≥ 4 cutoff. For classifying preserved or impaired cognition, SDMT standardized *Z*-scores were calculated based on German normative healthy control data for the Berlin cohort [33] and norm scores of matched healthy controls for the Amsterdam cohort [28]. PwMS reporting an SDMT *Z*-score below −1.5 were considered as cognitively impaired.

### Clinical worsening

Clinical worsening was assessed using the EDSS, 9HPT, T25FW, a combination of EDSS, 9HPT, and T25FW (EDSS +), or SDMT. EDSS-based worsening was defined as an increase in EDSS of  ≥ 1*.*5 points for a baseline score of 0, an increase of  ≥ 1*.*0 for a baseline score between 1.0 and 5.5, or an increase of  ≥ 0*.*5 for a baseline EDSS score of  ≥ 6.0 [34]. For 9HPT and T25FW, clinically meaningful worsening was defined as a 20% increase in the time required to finish the test compared to the baseline measurement [35, 36]. For 9HPT, worsening in the non-dominant hand and dominant hand was assessed separately. Worsening on EDSS + was defined as worsening on  ≥ 1 of the three components (EDSS, T25FW, or 9HPT (dominant or non-dominant hand)) [21]. Lastly, SDMT worsening was defined as an increase of  ≥ 4 points compared to the baseline measure [37].

### Input features

To compare the performance of clinical and MRI-derived features for classifying clinical impairment and predicting clinical worsening, and assess which combination would result in the highest performing models, five different feature sets were defined: (1) clinical data, (2) global MRI volumes, (3) clinical data + global MRI volumes, (4) regional MRI volumes, (5) clinical data + regional MRI volumes. See Table 1 for an overview of included features in each feature set. Clinical data included age, sex, symptom duration, MS subtype (CIS, RRMS, or progressive MS), and use of DMT (yes or no).

**Table 1:** Combinations of clinical and MRI features for classifying clinical impairment and worsening

Feature set	No	Variables
Clinical data	5	Sex, age, symptom duration, MS subtype, DMT
Global MRI volumes	6	WBV, CGMV, DGMV, LVV, CCerV, T2LV
Clinical data + global MRI volumes	11	Sex, age, symptom duration, MS subtype, DMT, WBV, CGMV, DGMV, LVV, CCerV, T2LV
Regional MRI volumes	116	WBV, BNA-CGMV (*n* = 105), thalamus, accumbens, putamen, caudate, pallidum, amygdala, hippocampus, LVV, CCerV, T2LV
Clinical data + regional MRI volumes	121	Sex, age, symptom duration, MS subtype, DMT, WBV, BNA-CGMV (*n* = 105), thalamus, accumbens, putamen, caudate, pallidum, amygdala, hippocampus, LVV, CCerV, T2LV

*BNA-CGMV* Brainnetome Atlas regional cortical gray matter volumes; *CCerV* cortical cerebellum volume; *CGMV* cortical gray matter volume; *DGMV* deep gray matter volume; *DMT* disease-modifying therapy; *LVV* lateral ventricular volume; *T2LV* T2-lesion volume; *WBV* whole-brain volume

### Machine learning model training

Five ML algorithms were compared for classification of clinical impairment and prediction of worsening for the different clinical outcomes. A comparison of multiple ML models was conducted because these models have varying abilities to capture linear and non-linear relationships between input features [38]. As linear classifiers, logistic regression (LR) and support vector machine with a linear kernel (SVM-lin) [39] were selected due to their robust performance demonstrated in prior structural neuroimaging studies [40, 41]. Their performance was compared to three non-linear models that have been successfully applied in other studies [42]: SVM with a radial basis function kernel (SVM-RBF) [43], eXtreme Gradient Boosting Classifier (XGBoost) [44], and random forest (RF) [45]. The ML pipelines were implemented in Python 3.9.15 using the scikit-learn [46] and xgboost [47] software packages. Due to variations in demographics, follow-up times, and the use of different MRI scanners and protocols between the cohorts, it was unfavorable to employ one cohort as a training set and the other as a validation set. Consequently, machine learning models were independently trained on the data from each center. Preprocessing of all input features included standardization by removing the mean and scaling to unit variance [48]. In addition, random oversampling of the minority class was used in the preprocessing pipeline to account for class imbalance in model training. Due to the relatively low sample size of our data, stratified Monte–Carlo cross-validation with ten repetitions (i.e., 10 randomly selected test sets) was performed using an 80%/20% train/test split ratio to avoid evaluation bias resulting from sampling effects [49, 50]. A stratified 5-fold cross-validation was applied within the training set of each repetition for hyperparameter optimization [51]. Since not all clinical outcome variables were available for each participant, train and test sets were different for each clinical outcome due to random selection. Model performance was assessed using several metrics: area under the curve (AUC) of the receiver operating characteristics curve, balanced accuracy (BA), precision, and recall (or sensitivity). AUC assesses the model's ability to distinguish between classes by plotting the true positive rate against the false positive rate across all possible classification thresholds. Balanced accuracy represents the average of recall and specificity, providing a balanced view of the model's performance across both classes. Precision quantifies the proportion of true positives among all positive predictions, highlighting the model's accuracy in predicting positive instances. Recall, also known as sensitivity, measures the proportion of actual positives correctly identified by the model, indicating its effectiveness in detecting positive instances. The final performance of the models was ranked based on the average and standard deviation of the AUC across the 10 repetitions. Statistical significance of the best performing ML model for each outcome measure was determined using permutation testing [52]. To reduce computational time, the *p* value was derived based on 100 permutations.

### Model explanations

To understand which combination of clinical and MRI features were most relevant for clinical impairment and worsening predictions in pwMS, SHAP values were calculated in a post hoc analysis. SHAP is a local model explanation method aimed to explain the model prediction for each subject by computing the relative importance of every input feature for the final prediction [53]. SHAP values explain the difference between the individual prediction and the average prediction. For an individual, the sum of all SHAP values equals the difference between their prediction and the average probability of clinical impairment or worsening. Global ranking of feature importance was defined as the mean absolute SHAP value of each feature across all subjects and all test sets [54]. SHAP values were calculated with the KernelSHAP method implemented in Python.

## Results

### Demographics

**Fig. 2:**
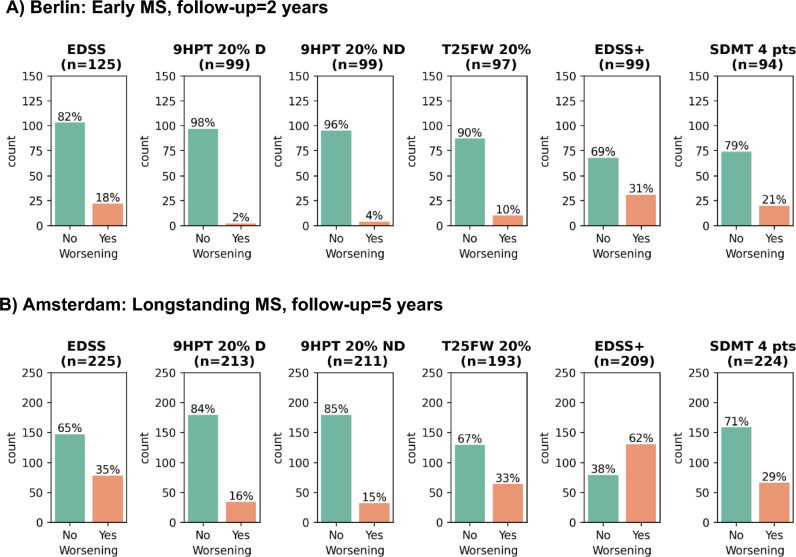
Number of subjects with clinical worsening based on multiple clinical end points for the **A** Berlin cohort and **B** Amsterdam cohort. The percentage of pwMS showing worsening (orange) or being stable (green) on a clinical outcome measure over the follow-up period varies for each outcome measure. The total sample size for each outcome measure (*n*) is displayed above the graphs. *EDSS* Expanded Disability Status Scale; *9HPT* 9-Hole Peg Test (*D* dominant hand; *ND* non-dominant hand); *SDMT* Symbol Digit Modalities Test; *T25FW* Timed 25-Foot Walk Test

A total of 125 participants from Berlin and 330 participants from Amsterdam were included. Demographic, clinical, and global MRI variables of the cohorts are shown in Table 2. The participants from Amsterdam were older compared to those from Berlin (47.7 ± 10.9 vs. 33.2 ± 7.2, *p* < 0.001), had a longer disease duration (14.8 ± 8.5 vs. 0.6 ± 0.7, *p* < 0.001), and a higher EDSS at baseline (3.0 [2.0 − 4.0] vs. 1.5 [1.0 − 2.0], *p* < 0.001). For the Berlin cohort only, 2% (*n* = 3) of pwMS had a high disability (EDSS ≥ 4) at baseline and 2% (*n* = 2) had cognitive impairment (SDMT Z ≤  − 1.5). For the Amsterdam cohort, 39% (*n* = 129) of pwMS had a high disability at baseline and 36% (*n* = 119) had cognitive impairment. See Fig. 2 for total sample sizes for each longitudinal clinical outcome measure and percentages of patients showing worsening during the follow-up period. For the Berlin cohort, a 2-year clinical follow-up was available for all 125 patients, of whom 18% (*n* = 22) showed worsening on EDSS and 21% (*n* = 20) on SDMT. For the Amsterdam cohort, a 5-year follow-up was available for 225 pwMS, of whom 35% (*n* = 78) showed worsening on EDSS and 29% (*n* = 66) on SDMT. The lowest progression rates were seen for the 9HPT in both cohorts, with 2% (dominant hand, *n* = 2) and 4% (non-dominant hand, *n* = 4) in the Berlin cohort, and 16% (dominant hand, *n* = 34) and 15% (non-dominant hand, *n* = 32) in the Amsterdam cohort.

**Table 2:** Baseline demographic and clinical variables of the studied MS cohorts

	Berlin: early MS	Amsterdam: long-standing MS
Subjects (*n*)	125	330
Subjects at follow-up (*n*)	125	225
Follow-up time (years) [mean ± SD]	1.8 ± 0.3	4.8 ± 0.8
Phenotypes *(CIS/RRMS/SPMS/PPMS)*	32*/*94*/* − */* −	− */*174*/*34*/*18
Disease-modifying therapy (no/yes)	86*/*39	212*/*118
Age (years) [mean ± SD]	33*.*2 ± 7*.*2	47*.*7 ± 10*.*9
Disease duration (years) [mean ± SD]	0*.*6 ± 0*.*7	14*.*8 ± 8*.*5
EDSS [median [IQR]]	1*.*5 [1*.*0 − 2*.*0]	3*.*0 [2*.*0 − 4*.*0]
EDSS ≥ 4 (*n*/total *n*, %)	3*/*125, 2%	129*/*330, 39%
SDMT [mean ± SD]	60 ± 12	50 ± 13
SDMT *Z* ≤ − 1*.*5 (*n*/total *n*, %)	2*/*118, 2%	119*/*328, 36%
WBV (fraction) [mean ± SD]	0*.*74 ± 0*.*03	0*.*72 ± 0*.*04
CGMV (fraction) [mean ± SD]	0*.*30 ± 0*.*02	0*.*32 ± 0*.*02
DGMV (fraction) [mean ± SD]	0*.*039 ± 0*.*003	0*.*034 ± 0*.*003
LVV (fraction) [mean ± SD]	0*.*011 ± 0*.*006	0*.*020 ± 0*.*011
T2LV (mL) [median [IQR]]	1*.*1 [0*.*3–2*.*7]	10*.*0 [4*.*9 − 19*.*1]

MRI volumes are expressed as a fraction of total intracranial volume and T2-lesion volumes in mL

*CIS* clinically isolated syndrome; *CGMV* cortical gray matter volume; *DGMV* deep gray matter volume; *EDSS* Expanded Disability Status Scale; *LVV* lateral ventricular volume; *MS* multiple sclerosis; *PPMS* primary progressive MS; *RRMS* relapsing-remitting MS; *SDMT* Symbol Digit Modalities Test; *SPMS* secondary progressive MS; *T2LV* T2-lesion volume; *WBV* whole-brain volume;  *SD* standard deviation; *IQR* interquartile range

### Classification of clinical impairment

**Fig. 3:**
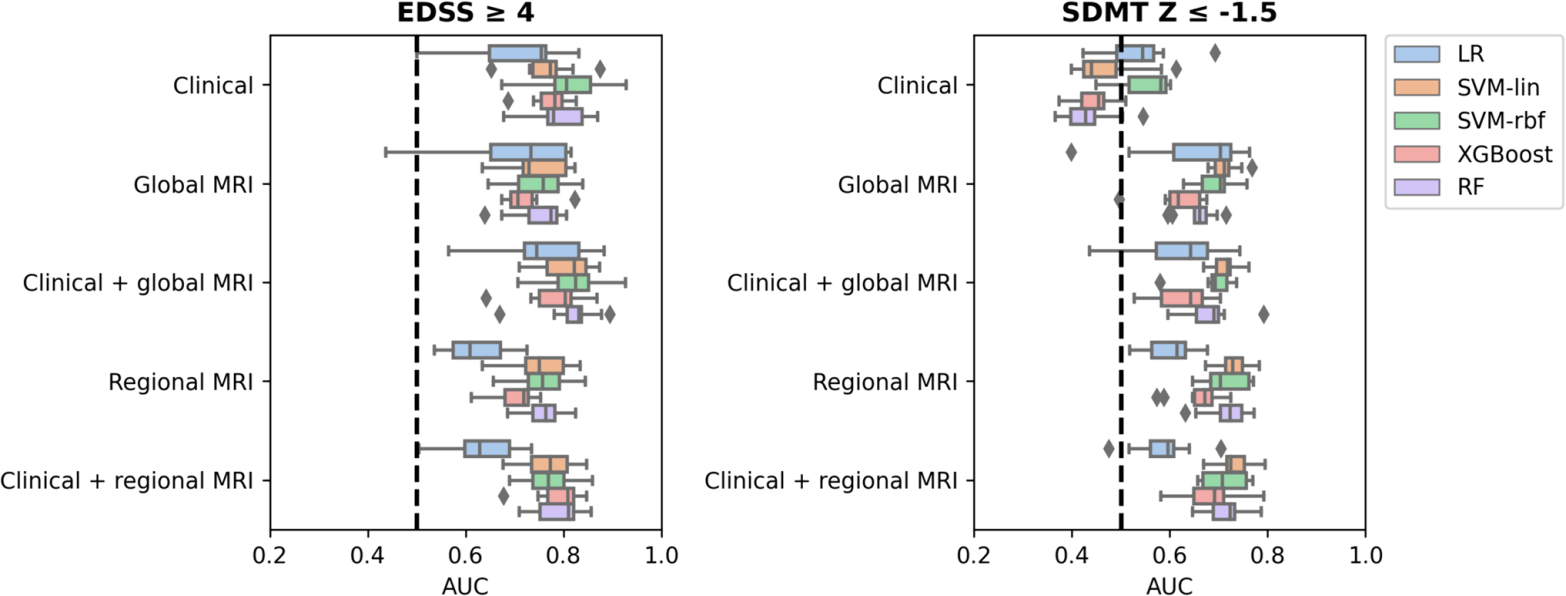
Performance of ML models in classifying clinical impairment at baseline across ten partitions (Amsterdam cohort, *n* = 330). For determining EDSS ≥ 4, the highest mean AUC was achieved by SVM-RBF with feature set Clinical + global MRI (AUC = 0*.*83 ± 0*.*07, *p* = 0.016), for determining SDMT Z ≤ 1*.*5 the highest mean AUC was found for SVM-lin with regional MRI as feature set (AUC = 0*.*73 ± 0*.*04, *p* = 0.008). *AUC* area under the curve; *EDSS* Expanded Disability Status Scale; *SDMT* Symbol Digit Modalities Test; *LR* logistic regression; *SVM-lin* support vector machine with a linear kernel; *SVM-RBF* SVM with a radial basis function kernel; *XGBoost* eXtreme Gradient Boosting Classifier; *RF* random forest

Due to the low proportion of pwMS having clinical impairment in the Berlin cohort (2% of pwMS had a high disability and 2% had cognitive impairment), cross-sectional clinical impairment classification was only performed for the Amsterdam cohort. The best performance for determining high disability at baseline was achieved by the clinical + global MRI feature set (AUC = 0*.*83 ± 0*.*07, BA = 0.76 ± 0.09, precision = 0.68 ± 0.11, recall = 0.74 ± 0.11, *p* = 0.015) using SVM-RBF. As shown in Fig. 3, all models showed good performance for determining high disability at baseline (mean AUC 0.75–0.78 across all feature sets), with the lowest performance for LR (mean AUC = 0.68). The model performance for determining impaired cognition was the highest for SVM-lin with regional MRI volumes as input feature set (AUC = 0*.*73 ± 0*.*04, BA = 0.67 ± 0.03, precision = 0.60 ± 0.11, recall = 0.55 ± 0.09, *p* = 0.008). Clinical features only could not predict cognitive impairment (SVM-RBF: AUC = 0*.*55 ± 0*.*05, *p* = 0.27).

### Important brain regions for determining clinical impairment

**Fig. 4:**
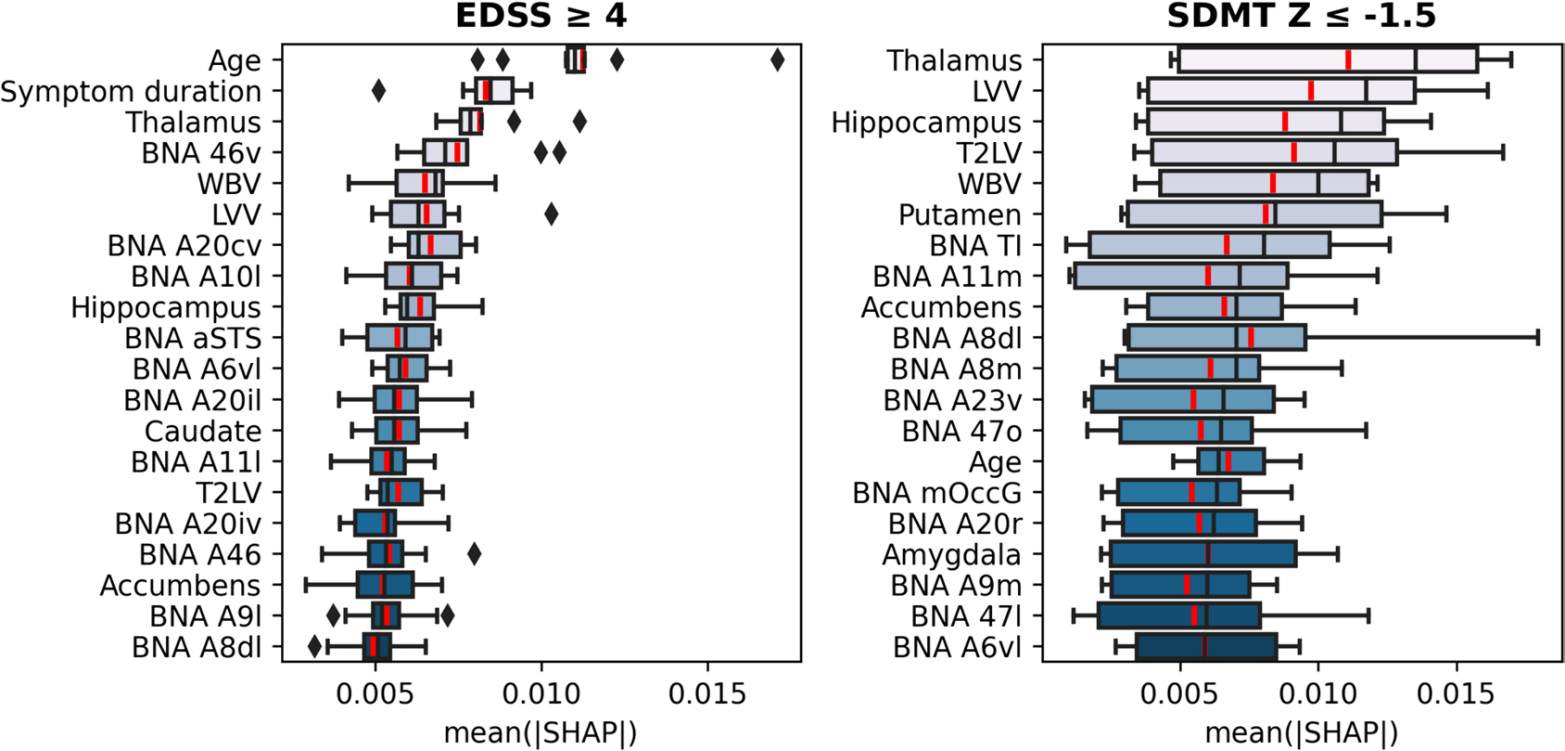
Distributions of SHAP feature importance for the 20 most important features using SVM-RBF in determining high disability (left, AUC = 0.77 ± 0.05, *p* = 0.035) and cognitive impairment (right, AUC = 0.72 ± 0.04, *p* = 0.008) at baseline (Amsterdam cohort, *n* = 330). The mean of the distributions is shown in red. The Brainnetome Atlas (BNA) cortical regions are plotted in Fig. 5 and described in Table 3. *EDSS* Expanded Disability Status Scale; LVV lateral ventricular volume; *SDMT* Symbol Digit Modalities Test; T2LV T2-lesion volume; WBV whole-brain volume

**Fig. 5:**
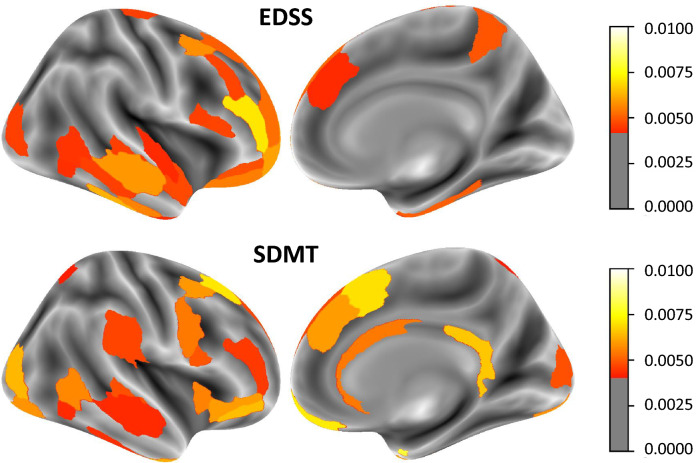
Most important brain regions for classifying clinical impairment using SVM-RBF for determining high disability (left) and cognitive impairment (right) at baseline (Amsterdam cohort, *n* = 330). Intensity represents the median SHAP value across all ten partitions. *EDSS* Expanded Disability Status Scale; *SDMT* Symbol Digit Modalities Test

We used SHAP values to assess which clinical features and brain regions were most important for classifying high disability and impaired cognition in long-standing MS (Amsterdam cohort). The most informative features were determined for the SVM-RBF model since it had the highest average performance across all feature sets in determining high disability (AUC = 0*.*78 ± 0*.*07) and impaired cognition (AUC = 0*.*67 ± 0*.*08). Figure 4 presents the 20 most important features from the feature set including all clinical and MRI features (clinical data + regional MRI volumes). Based on all input features, the SVM-RBF model achieved an AUC of 0*.*77 ± 0*.*05 (*p* = 0.035) in determining high disability and an AUC of 0*.*72 ± 0*.*04 (*p* = 0.008) in determining cognitive impairment. The feature importance for the other feature sets are shown in Supplementary Figs. 1 and 2. Most relevant features for determining high disability were age, disease duration, LVV, DGM volumes (thalamus, hippocampus, caudate, and accumbens) and CGM regions in the middle frontal gyrus (BNA: A46, A46v, A6vl, A10l) and inferior temporal gyrus (BNA: A20cv, A20il), see Fig. 5 and Table 3. For determining cognitive impairment, the most relevant features were regional MRI volumes, including DGM volumes (thalamus, hippocampus, putamen, accumbens), LVV, T2LV, and volumes of the superior frontal gyrus (BNA: A8dl, A8m), orbital gyrus (BNA: A11m), and parahippocampal gyrus (BNA: TI).

**Table 3:** Most important cortical regions within the Brainnetome Atlas (BNA) determining high disability and cognitive impairment

BNA region	EDSS	
	Gyrus	Lobe
A9/46v, ventral area 9/46	MFG, middle frontal gyrus	Frontal lobe
A20cv, caudoventral of area 20	ITG, inferior temporal gyrus	Temporal lobe
A10l, lateral area 10	IFG, inferior frontal gyrus	Frontal lobe
aSTS, anterior superior temporal sulcus	MTG, middle temporal gyrus	Temporal lobe
A6vl, ventrolateral area 6	MFG, middle frontal gyrus	Frontal lobe
A20il, intermediate lateral area 20	ITG, inferior temporal gyrus	Temporal lobe
A11l, lateral area 11	OrG, Orbital Gyrus	Frontal lobe
A20iv, intermediate ventral area 20	ITG, inferior temporal gyrus	Temporal lobe
A46, area 46	MFG, middle frontal gyrus	Frontal lobe
A9l, lateral area 9	SFG, Superior Frontal Gyrus	Frontal lobe
A8dl, dorsolateral area 8	SFG, superior frontal gyrus	Frontal lobe

BNA region	SDMT	
	Gyrus	Lobe
TI, area TI (temporal agranular insular cortex)	PhG, parahippocampal gyrus	Temporal lobe
A11m, medial area 11	OrG, orbital gyrus	Frontal lobe
A8dl, dorsolateral area 8	SFG, superior frontal gyrus	Frontal lobe
A8m, medial area 8	SFG, superior frontal gyrus	Frontal lobe
A23v, ventral area 23	CG, cingulate gyrus	Limbic lobe
A12/47o, orbital area 12/47	OrG, orbital gyrus	Frontal lobe
mOccG, middle occipital gyrus	LOcC, lateral occipital cortex	Occipital lobe
A20r, rostral area 20	ITG, inferior temporal gyrus	Temporal lobe
A9m, medial area 9	SFG, superior frontal gyrus	Frontal lobe
A12/47l, lateral area 12/47	OrG, orbital gyrus	Frontal lobe
A6vl, ventrolateral area 6	MFG, middle frontal gyrus	Frontal lobe

BNA Brainnetome Atlas; *EDSS* Expanded Disability Status Scale; *SDMT* Symbol Digit Modalities Test

### Prediction of clinical worsening

The performance of the ML models in predicting clinical worsening was evaluated based on the mean AUC, BA, precision, and recall for each longitudinal outcome measure across all test sets. The combination of the best ML models and feature sets for each prediction task are presented in Table 4. For the Berlin cohort, prediction of 9HPT 20% worsening could not be assessed due to a large class imbalance (2% worsening on dominant hand and 4% worsening on non-dominant hand). Although the prediction of EDSS worsening over a 2-year follow-up showed the highest AUC using SVM-RBF and global MRI volumes as input features (AUC = 0*.*73 ± 0*.*13), precision was low (0*.*24 ± 0*.*09) and the prediction was not significant after permutation testing (*p* = 0.163). Other measures of clinical worsening also did not reach significance after permutation testing, with mean AUC varying from 0*.*53 to 0*.*73 and mean BA between 0.51 and 0.63. For the Amsterdam cohort, clinical worsening could not be predicted over the 5-year follow-up, as none of the ML models reached significance after permutation testing. The highest prediction performance was achieved for 9HPT 20% worsening in the dominant hand (AUC = 0.63 ± 0.11, BA = 0.53 ± 0.08), with low precision (0.21 ± 0.12) and recall (0.23 ± 0.17) values. The ML models for predicting other clinical worsening outcomes reached a mean AUC between 0.54 and 0.63, with a mean BA between 0.50 and 0.59.

**Table 4:** Classification metrics of best ML models and combination of clinical and MRI features predicting clinical worsening on randomly sampled test sets

Berlin, early MS (follow-up: 2 years)						
Outcome	Best ML model	Best feature set	AUC	BA	Precision	Recall
EDSS	SVM-RBF	Global MRI	0.73 ± 0.13	0.62 ± 0.15	0.24 ± 0.09	0*.*58 ± 0*.*33
T25FW 20%	SVM-lin	Regional MRI	0.53 ± 0.22	0.51 ± 0.16	0.08 ± 0.13	0*.*22 ± 0*.*36
EDSS +	XGB	Clinical + global MRI	0.66 ± 0.11	0.60 ± 0.14	0.46 ± 0.22	0*.*47 ± 0*.*19
SDMT 4 pts	SVM-lin	Regional MRI	0.67 ± 0.11	0.63 ± 0.15	0.29 ± 0.17	0*.*64 ± 0*.*36

Amsterdam, long-standing MS (follow-up: 5 years)						
Outcome	Best ML model	Best feature set	AUC	BA	Precision	Recall
EDSS	RF	Clinical	0.57 ± 0.10	0.54 ± 0.06	0.40 ± 0.07	0.41 ± 0.16
9HPT 20% D	RF	Clinical	0.63 ± 0.11	0.53 ± 0.08	0.21 ± 0.12	0.23 ± 0.17
9HPT 20% ND	SVM-RBF	Global MRI	0.57 ± 0.12	0.59 ± 0.12	0.24 ± 0.11	0.44 ± 0.23
T25FW 20%	LR	Clinical	0.59 ± 0.08	0.57 ± 0.07	0.39 ± 0.07	0.63 ± 0.17
EDSS +	SVM-RBF	Clinical	0.60 ± 0.07	0.59 ± 0.08	0.71 ± 0.07	0.61 ± 0.05
SDMT 4 pts	LR	Clinical + global MRI	0.54 ± 0.13	0.50 ± 0.11	0.34 ± 0.27	0.39 ± 0.31

Mean values and standard deviation of metrics were calculated on ten test sets. None of the classifiers reached significance after permutation testing

*EDSS* Expanded Disability Status Scale; *T25FW 20%* 20% worsening in Timed 25-Foot Walk Test; *EDSS* + worsening based on EDSS + ; *SDMT 4 pts* worsening of 4 points in Symbol Digit Modalities Test; *9HPT 20% D/ND* 20% worsening in 9-Hole Peg test for dominant/non-dominant hand; *AUC* area under the curve; *BA* balanced accuracy

## Discussion

In this study, five different ML models were applied to determine the clinical impairment and predict clinical worsening in pwMS based on clinical and structural MRI input features. For baseline models, the classification of high disability and cognitive impairment exhibited commendable accuracy when relying on a combination of clinical and regional MRI input features. Nevertheless, forecasting disease worsening in pwMS over a longitudinal period of 2–5 years, utilizing baseline clinical and structural MRI features, did not yield significant prediction accuracies.

In the classification of high versus low disability at baseline, both clinical characteristics and MRI volumes played a crucial role in the prediction, achieving an AUC of 0.83. However, for cognitive impairment, regional MRI volumes were most important, as clinical features alone offered little explanatory value. The performance of the cognitive classification task (AUC = 0.73) was highly comparable to another study predicting cognitive impairment in a large cohort (*n* = 540), achieving an AUC of 0.74 using MRI features as input only [55]. From all MRI features, the thalamus volume was the most important regional MRI feature associated with both disability and cognitive impairment, which is in line with previous findings [9, 56, 57]. Regarding other DGM volumes, atrophy of the hippocampus and accumbens was important for determining both high disability and cognitive impairment, while caudate volume seemed more predictive for disability, and the volume of the amygdala and putamen for cognitive impairment. The classification tasks demonstrated comparable performance whether utilizing global MRI volumes or intricate parcellations of the CGM with the BNA. The advantage of the latter approach is that distinct CGM regions could be identified that were important for high disability and cognitive impairment. Consistent with previous work using other statistical methods, mostly regions in the temporal and frontal gyrus were important for determining high disability and cognitive impairment [58, 59]. From the temporal lobe, specific areas in the inferior and middle temporal gyrus were most important for EDSS, while the parahippocampal gyrus was more important for determining cognitive impairment. When comparing the stability of feature importance across tasks, it is important to highlight that the feature importance for predicting cognitive impairment were less consistent across various test splits. This reduced stability might be attributed to the lower task performance (0.73 compared to 0.83). However, it could also suggest that the model captures heterogeneous cognitive profiles in pwMS, since the SDMT assesses various cognitive processes [60].

Despite achieving good performance in cross-sectional classification tasks, predicting future disease worsening over 2 and 5 years proved to be unattainable using the same ML models and input variables. While two earlier ML studies reported more promising results in predicting MS disease worsening, these results were limited by relying on relatively small sample sizes and model evaluation on only one small test set, possibly reporting overly optimistic results [15, 61]. In larger cohort studies using similar ML approaches, baseline data had limited predictive value for disease worsening over 5 years, while incorporating longitudinal observations of clinical and brain MRI changes in the first two years improved prediction performance (AUC = 0.75–0.83) [13, 62]. Furthermore, incorporating standardized disease history from electronic health records in combination with MRI might be a promising direction to increase data availability and information content with higher frequency for improving ML prediction models [63]. In addition, all previous ML studies investigating disease worsening in MS used EDSS as the clinical end point. While EDSS-based definitions are still considered the gold standard in clinical research, they are often criticized for low sensitivity and reliability [64]. To address the challenge of heterogeneous subjective scoring in clinical assessments, one promising approach involves leveraging ML models capable of accommodating label uncertainty [65]. In our study, we took a different approach by exploring a range of other widely used clinical end points, including assessments of hand dysfunction (9HPT), walking dysfunction (T25FW), and a combination of EDSS, 9HPT, and T25FW (EDSS +). However, we did not observe an improvement in the prediction performance for all these outcomes. While 9HPT and T25FW have a lower measurement error compared to the EDSS, worsening could still not be predicted by baseline MRI measures [22]. These findings highlight the need for defining accurate clinical end points in addition to the exploration of better predictors [66]. In addition to predicting disability worsening, SDMT worsening of at least 4 points over 5 years was defined as a cutoff for cognitive worsening, but also did not achieve significant prediction performance. This is in contrast to an earlier investigation of the Amsterdam MS cohort, which showed that cortical atrophy was predictive of cognitive decline, using traditional logistic regression and an extensive cognitive test battery to define cognitive worsening [30]. The difference in predictive performance may be explained by the lower sensitivity of the SDMT as a standalone measure to capture cognitive decline [67, 68].

The main limitation of our study is the relatively small sample size of our data sets for an ML-based analysis, along with the lack of evaluation on an additional external test set. Despite the application of our approach across two diverse cohorts that span early to late stages of MS, the divergent data collection and follow-up times (2 vs 5 years) prevented the merging of cohorts for analysis and the utilization of one cohort as an independent test set. Future ML studies with larger datasets are required to explore individualized prediction of disease course both in early and later MS phases, facilitating early detection of high-risk patients and enabling personalized treatment strategies. Predictive variables for disease progression may differ between MS disease stages, as there are some indications that some predictors such as white matter damage are predictive for worsening in early stages and other measures such as cortical atrophy are specific for later, more progressive, stages of MS [30]. Furthermore, considering the predictive value of spinal cord lesions and atrophy is crucial, as these factors significantly contribute to walking dysfunction in long-standing disease [69]. In addition, the limited sample size resulted in a low stability of feature importance across the evaluated test sets, especially for classifying cognitive impairment. To establish a more robust understanding of the relationship between clinical and structural brain information and clinical end points related to disease severity and progression, further investigations with larger sample sizes and extended follow-up durations are essential. Lastly, the currently used clinical end points may not be specific enough to capture disease progression in MS. Development of new outcome measures, such as frequent smartphone-based assessments, may be more sensitive in capturing worsening in various symptoms of MS [70].

## Conclusion

While ML models could accurately identify patients with clinical impairment based on clinical data and regional MRI volumes, accurate prediction of MS disease worsening remains an unmet need. Improving clinical end point metrics and using larger standardized datasets representative of the heterogeneity in MS can be a promising direction for future ML research.

### Supplementary Information

Below is the link to the electronic supplementary material.

Supplementary file1 (DOCX 451 KB)

## Data Availability

Anonymized data can be shared upon reasonable request from a qualified investigator.
