# Supplementary material for: Evaluation of machine learning-based classification of clinical impairment and prediction of clinical worsening in multiple sclerosis
Source: J Neurol. 2024 Jun 23;271(8):5577–89. doi: 10.1007/s00415-024-12507-w (PMC11319410; doi:10.1007/s00415-024-12507-w)
Supplement: Supplementary file 1 — Supplementary file1 (DOCX 451 KB) [file 415_2024_12507_MOESM1_ESM.docx]

### Supplementary materials


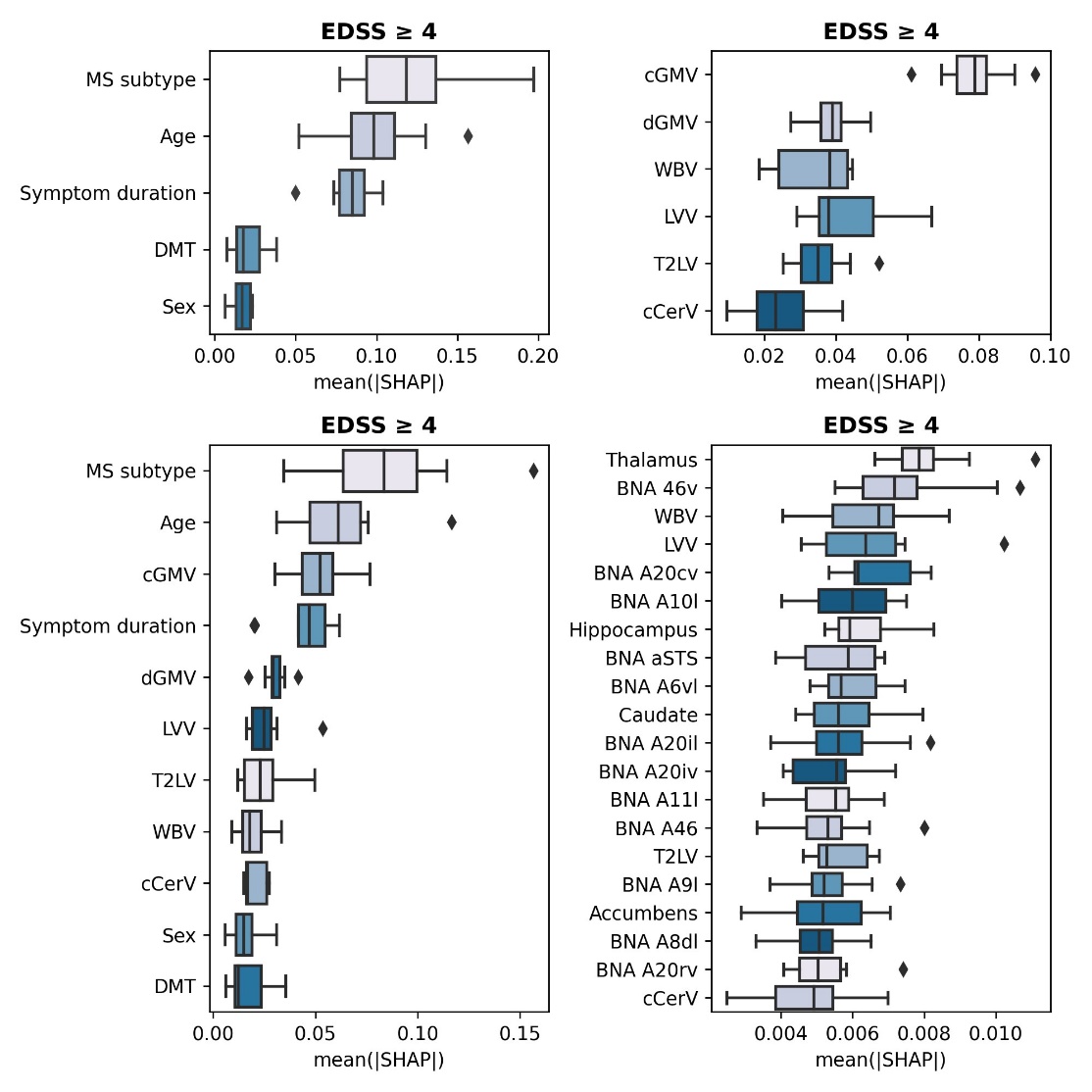


**Figure 1**. Prediction high disability using SVM-rbf. Distributions of SHAP feature importances for each feature set: 1) Clinical (AUC=0*.*81±0*.*07, *p*=0.018) 2) global MRI (AUC=0*.*75±0*.*06, *p*=0.043) 3) clinical + global MRI (AUC=0*.*83±0*.*06, *p*=0.036) 4) regional MRI (AUC=0*.*76±0*.*05, *p*=0.035).


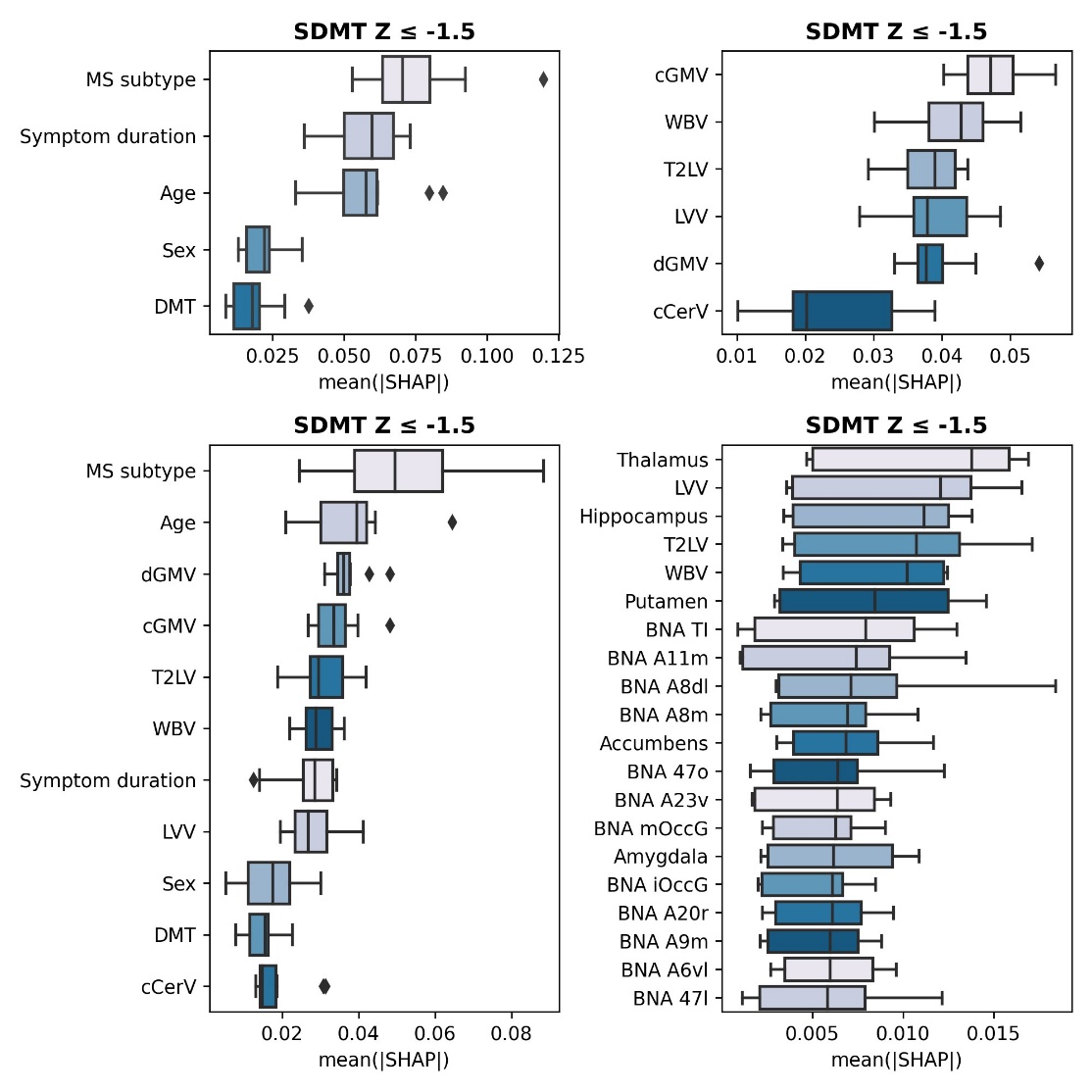


**Figure 2.** Prediction cognitive impairment using SVM-rbf. Distributions of SHAP feature importances for each feature set: 1) Clinical (AUC=0*.*55±0*.*05, *p*=0.27) 2) global MRI (AUC=0*.*69±0*.*04, *p*=0.057) 3) clinical + global MRI (AUC=0*.*69±0*.*04, *p*=0.041) 4) regional MRI (AUC=0*.*72±0*.*04, *p*=0.008).
